# Surgical treatment of clinically infected mandibular fractures

**DOI:** 10.1007/s10006-024-01213-6

**Published:** 2024-01-30

**Authors:** Marko Oksa, Aleksi Haapanen, Leena Kannari, Jussi Furuholm, Johanna Snäll

**Affiliations:** 1 Department of Oral and Maxillofacial Diseases, University of Helsinki, Haartmaninkatu 1, FI-00290 Helsinki, Finland; 2 Helsinki University Hospital, Haartmaninkatu 1A, FI-00029 Helsinki, Finland

**Keywords:** Mandible, Fracture, Infection, Surgery

## Abstract

**Purpose:**

To clarify reasons for infections, surgical techniques, and occurrence of postoperative surgical site complications in infected mandibular fractures.

**Methods:**

Patients with clinically infected mandibular fracture of the dentate part without preceding surgery were studied retrospectively. Clinical infection was defined to occur if pus, abscess, or a fistula in the fracture area was present. Patient-, fracture-, and surgery-related variables were evaluated, and predictors for postoperative complications were analysed.

**Results:**

Of 908 patients with surgically treated fracture in the dentate part of the mandible, 41 had infected fracture at the time of surgery (4.5%). Of these patients, 46.3% were alcohol or drug abusers. Median delay from injury to surgery was 9 days. Patient-related factors were the most common cause for delayed surgery (n = 30, 73.2%), followed by missed diagnosis by a health care professional (n = 8, 19.5%). Twenty-two fractures were treated via extraoral approach (53.7%) and the remaining 19 intraorally (46.3%). Postoperative surgical site complications were found in 13 patients (31.7%), with recurrent surgical site infections predominating. Notable differences between total complication rates between intraoral and extraoral approaches were not detected. Secondary osteosynthesis for non-union was conducted for one patient treated intraorally.

**Conclusions:**

Postoperative surgical site complications are common after treatment of infected mandibular fractures, and these occur despite the chosen surgical approach. Infected mandibular fractures heal mainly without bone grafting, and non-union is a rare complication. Due to the high complication rate, careful perioperative and postoperative care is required for these patients.

## Introduction

Fractures of the dentate part of the mandible are prone to infections, especially as a postoperative complication of surgical management [1–4]. However, sometimes the fracture is already clinically infected at the time of surgery. The main cause for the infected fracture is reported to be a delay in seeking treatment due to a low level of patient compliance, injury during intoxication, and lack of understanding of the consequences of the fracture [5].

The aim in surgical treatment of an infected fracture of the mandible is to restore occlusion, make the patient pain-free, and eliminate the infection. Historically, the management of infected mandibular fracture was conducted with maxillomandibular fixation, external fixators, and/or splints [6]. Gradually, rigid internal fixation (RIF) has replaced the previous protocol, and fracture stabilization with RIF combined with abscess incision and drainage, debridement, and reduction of the fracture has been found to be successful [6, 7]. In addition, prophylactic antibiotic medication as part of the treatment of mandible fractures is compulsory [8] and in infected fractures is indicated and should be targeted more precisely after specimen culturing [7].

Mandibular fractures can be treated surgically intraorally or extraorally. No difference between complication rates has been found between different surgical approaches in a previous retrospective study [9]. Infected mandibular fractures are rare, and therefore, low numbers of patients have been included in earlier studies [6, 7, 10–13]. Due to heterogeneity in fracture types, criteria for infection, and surgical approaches, clear guidelines for treatment of infected mandibular fractures are lacking. Thus, we clarified reasons for infections, surgical techniques, and occurrence of postoperative surgical site complications in these fractures.

## Materials and methods

### Data collection

Data on patients with surgically treated mandibular fractures from recent injury who were treated at Helsinki University Hospital, Helsinki, Finland between 2012 and 2022 were collected retrospectively.

### Inclusion criteria

Patients with infected fracture of the mandible without preceding surgery were included in the study. To be infected, one or more of the following signs must be detected: pus formation in the fracture site, abscess, and/or draining fistula (intraoral or extraoral).

### Study variables

Patient-, fracture-, and surgery-related variables were evaluated, and rate of and predictors for postoperative surgical site complications were analysed. Patient-related factors included age, sex, smoking status, alcohol and drug abuse, injury mechanism, treatment delay from accident to surgery in days, and factors contributing to delayed surgery. Fracture-related factors were fracture site and comminution of the fracture. Additionally, fractures were divided into mildly and severely infected groups. Fracture comminution was assessed after the review of dental panoramic radiographs and computed tomography images by authors A.H. and J.S. A fracture was defined as minor comminution if there was one or more fragments larger than the size of the crown of a premolar not involving the full vertical height of the mandibular arch. A comminution was major when one or more small or large intermediate fragment(s) were involved the full height of the mandibular arch [14]. Severely infected fractures included cases with abscess, cellulitis, and/or extraoral fistula. Fractures with local pus formation and/or intraoral fistula were classified as mildly infected. Surgery-related factors were fixation method, surgical approach, bone grafting, and use of a drain. Additionally, duration of postoperative antibiotic course, delay from surgery to postoperative surgical site complication in days, and occurrence of postoperative surgical site complications were recorded.

### Surgical technique and antibiotic therapy

Fracture stabilization was combined with drainage and debridement of the infected fracture site. Reconstruction (2.3 mm) and non-reconstruction (2.0 mm) plates with or without lag screw(s) were used. Antibiotic treatment was started when the patient entered the hospital. Antibiotics were administered in anaesthesia induction, and postoperative antibiotic therapy was prescribed for all patients and in most cases targeted according to the result of bacterial culture.

### Statistical analysis

Statistical analyses were performed with a statistical software package (SPSS for Macintosh version 28, IBM). Differences between study groups were assessed with t test for normally distributed and Mann–Whitney U test for non-normally distributed continuous variables. Categorical variables were cross-tabulated and analysed with Pearson chi-squared test or Fisher’s exact test if expected values were below 5. P values < 0.05 were considered significant throughout the analyses.

## Results

During the 11-year research period, infected mandibular fractures were located only in the dentate part of the mandible. Of 908 patients with fracture in the dentate part of the mandible treated surgically, 41 had clinically infected fracture (4.5%) and were included in this study. None of the patients were edentulous. Most of the patients were male (n = 35, 85.4%). Physical violence was the most common cause of fracture (n = 22, 53.7%). Alcohol and/or drug abuse occurred in about half of the patients (46.3%). All fractures were caused by blunt injury mechanism. None of the patients had fracture-associated soft tissue deficiency. Median follow-up time was 90 (range 1–1008) days. Treatment delay from accident to surgery varied widely with a median of 9 days. Patient-related factors were the most common reason for delayed surgery (n = 30, 73.2%). Delay caused by health care professionals for missed fracture was found in 8 patients (19.5%). Non-surgical treatment decision preceded infection in three patients (Table 1). Symphysis and parasymphysis covered 46.3% (n = 19) of all fracture sites. Specimen culturing was conducted in 33 patients (80.5%). Viridans streptococci was the most common group present in the cultures (13/33, 39.4%), and *Streptococcus anginosus* was identified in 8 patients (24.2%).

**Table 1:** Demographics, substance abuse, and fracture and infection aetiology in patients with infected mandibular fracture

All patients, n	41	
Age, years		
Range	17–73	
Mean	41	
Median	38	
Treatment delay from injury to surgery, days*		
Range	2–76	
Mean	16	
Median	9	
	**n**	**% of 41 patients**
Sex		
Male	35	85.4
Female	6	14.6
Smoking		
Yes	24	58.5
No	17	41.5
Alcohol and/or drug abuse		
Yes	19	46.3
No	22	53.7
Injury mechanism		
Assault	22	53.7
Traffic accident	1	2.4
Falling on the ground	14	34.1
Struck by an object	4	9.8
Factors contributing to delayed surgery		
Patient-related factors		
Yes	30	73.2
No	11	26.8
Fracture not detected at first visit to health care professional		
Yes	8	19.5
No	33	80.5
Initially planned non-surgical treatment, fracture infected during follow-up		
Yes	3	7.3
No	38	92.7

^*^Exact date of injury is missing in three cases (excluded from analyses)

Twenty patients (48.8%) had severe infection. Duration of postoperative antibiotic course ranged from 2 to 33 days with a median of 11 days. Extraoral approach was chosen in 53.7% of the fractures (n = 22). Table 2 shows differences in fracture- and treatment-related variables according to approach. Extraoral approach was associated with a longer treatment delay (*p* = 0.012). Reconstruction plates were solely used in surgeries with extraoral approach (*p* =  < 0.001), and drain usage was more common in patients treated extraorally (*p* = 0.014). Locking screws were used with all reconstruction plates with or without non-locking screws. A pre-bent plate was used in one surgery. One patient received bone grafting from the iliac crest in the primary fracture surgery.

**Table 2:** Patient-, fracture-, and treatment related variables in intraorally and extraorally treated patients

	Intraoral approach	Extraoral approach			P
All patients, n (%)	19 (46.3%)	22 (53.7%)			
Age, years					
Range	17–60	20–73			0.163
Mean	37.3	44.1			
Median	35	44			
Treatment delay from injury to surgery, days*					
Range	2–26	3–76			0.012**
Mean	8.5	19.9			
Median	6	10			
Duration of postoperative antibiotic course, days					
Range	2–15	2–33			
Mean	9.6	12.7			0.077
Median	10	12			
	**n**	**% of n**	**n**	**% of n**	
Smoking					
Yes	9	37.5	15	62.5	0.177
No	10	58.8	7	41.2	
Alcohol and/or drug abuse					
Yes	7	36.8	12	63.2	0.257
No	12	54.5	10	45.5	
Fracture site					
Symphysis/parasymphysis	10	52.6	9	47.4	0.509
Body	5	55.6	4	44.4	
Angle	4	30.8	9	69.2	
Comminution of the fracture					
Non-comminuted	16	48.5	17	51.5	0.703
Comminuted	3	37.5	5	62.5	
	**n**	**% of comminuted fractures**	**n**	**% of comminuted fractures**	
Minor comminution	2	50.0	2	50.0	1.000
Major comminution	1	25.0	3	75.0	
	**n**	**% of n**	**n**	**% of n**	
Infection severity					
Mild	13	61.9	8	38.1	0.062
Severe	6	30.0	14	70.0	
Fixation method					
Reconstruction plate	0	0	15	100.0	< 0.001**
Non-reconstruction plate	19	86.4	3	13.6	
Combined	0	0	4	100.0	
Bone grafting					
Yes	0	0	1	100	1.000
No	19	47.5	21	52.5	
Use of drain					
Yes	2	16.7	10	83.3	0.014**
No	17	58.6	12	41.4	

*Exact date of injury is missing in three cases (excluded from analyses)

^**^Statistically significant

Postoperative surgical site complication was observed in 13 patients (31.7%). Table 3 shows differences in variables between patients with and without postoperative complication. No significant differences were found between the groups. Lag screws were combined with plates in one surgery with intraoral approach. One patient had type 1 diabetes and had no postoperative complications. Other immunosuppressive conditions including immunosuppressive disorder and/or drug therapy were not detected.

**Table 3:** Fracture- and treatment related variables in patients with and without surgical site complication

	Surgical site complication present		Surgical site complication absent		P
All patients, n (%)	13 (31.7%)		28 (68.3%)		
Age, years					
Range	21–72		17–73		0.488
Mean	40.9		41.0		
Median	35		38		
Treatment delay from injury to surgery, days*					
Range	3–76		2–45		0.505
Mean	14.7		14.6		
Median	6		9		
Duration of postoperative antibiotic course, days					
Range	7–19		2–33		0.688
Mean	11.6		11.1		
Median	11		11		
	**n**	**% of n**	**n**	**% of n**	
Smoking					
Yes	8	33.3	16	66.7	0.790
No	5	29.4	12	70.6	
Alcohol and/or drug abuse					
Yes	6	31.6	13	68.4	0.987
No	7	31.8	15	68.2	
Fracture site					
Symphysis/parasymphysis	6	31.6	13	68.4	0.238
Body	1	11.1	8	88.9	
Angle	6	46.2	7	53.8	
Comminution of the fracture					
Non-comminuted	10	30.3	23	69.7	0.692
Comminuted	3	37.5	5	62.5	
	**n**	**% of comminuted fractures**	**n**	**% of comminuted fractures**	
Minor comminution	1	25.0	3	75.0	1.000
Major comminution	2	50.0	2	50.0	
	**n**	**% of n**	**n**	**% of n**	
Infection severity					
Mild	7	33.3	14	66.7	0.819
Severe	6	30.0	14	70.0	
Fixation method					
Reconstruction plate	5	33.3	10	66.7	1.000
Non-reconstruction plate	7	31.8	15	68.2	
Combined	1	25.0	3	75.0	
Bone grafting					
Yes	1	100.0	0	0	0.317
No	12	30.0	28	70.0	
Use of drain					
Yes	4	33.3	8	66.7	1.000
No	9	31.0	20	69.0	

^*^Exact date of injury is missing in three cases (excluded from analyses)

**Fig. 1:**
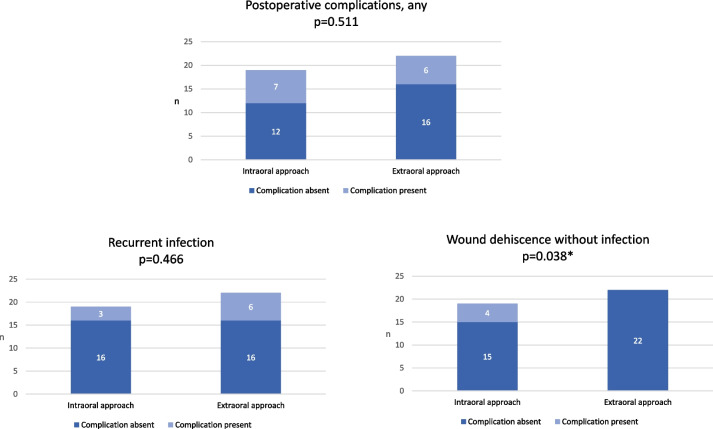
Postoperative surgical site complications in intraorally and extraorally treated patients with infected mandibular fracture. Wound dehiscence without infection occurred solely with intraoral approach (21.1%, *p* = 0.038). *Statistically significant

The occurrence range of postoperative complications was wide in both approaches: in intraorally operated patients from 7 to 129 days (median 28 days, mean 34.2 days) and in extraorally operated patients from 4 to 93 days (median 17 days, mean 31.7 days), *p* = 0.445. Recurrent infection was the most common complication (69.2%). Wound dehiscence without infection occurred solely with intraoral approach (21.1%, *p* = 0.038), whereas recurrent infections were slightly more often related to extraoral approach (27.3%) without statistical significance (Fig. 1). Secondary surgery and osteosynthesis due to non-union and recurrent infection were performed for one patient with intraoral approach. Plate(s) was removed due to recurrent infection or wound dehiscence in five patients (38.5% of patients with postoperative surgical site complication).

## Discussion

We evaluated treatment and surgical site complications of infected mandibular fractures. Altogether 4.5% of patients with fracture in the dentate part of the mandible had a clinically infected fracture at the time of surgery in this single-centre study covering a time span of 11 years. Postoperative surgical site complications were common in this subset of patients, afflicting 31.7% of cases. Many of these occurred with a notable delay of up to four months, emphasizing the importance of postoperative follow-up. Infected fractures can be selectively treated intraorally, especially if the infection is mild, but due to the risk of wound dehiscence, careful patient selection is required. Clinicians most often chose the extraoral approach in severely infected fractures and in cases with major comminution. However, infection complications occur regardless of the approach. Nevertheless, severe postoperative infections and non-union are rare.

Patient compliance-related factors were the most notable cause for delayed surgery (73.2%). Substance and alcohol abuse was quite common in this patient population (46.3%), which might cause the lack of understanding of the consequences of injury and explain the delay in seeking treatment. In our previous study in the same department, substance and alcohol abuse was lower among mandibular fracture patients in general (23.4%) [3]. However, 19.5% had a previous assessment with a health care professional after the injury, but the fracture was not detected and became infected later. In recent studies, mandibular fractures were missed in 20% of patients aged over 60 years [15] and in 14.8% of children (< 20 years) [16] during patients’ first health care assessment. Fractures were missed more frequently in elderly patients who lived in nursing homes or were in hospital at the time of injury and in patients aged under 13 years. Thus, careful clinical examination and imaging examinations (dental panoramic radiograph and/or computed tomography) are strongly recommended for all patients with recent mandibular injury.

Surgical site healing may be affected by local circumstances in the fracture site, such as severity of infection, atrophic mandible, patient-related factors, and the surgeon’s experience and preferences. Additionally, a recent study showed that severe external wounds increase the incidence of overall complication and infections[17]. No patients in this study were edentulous; thus, the presence of teeth and occlusion may have contributed to infection. As presented in our results, the clinicians choose the approach and osteosynthesis technique according to the severity of infection and fracture comminution. Reconstruction plate with or without combined non-reconstruction plate was used in 19 patients (46.3%), and these patients had a complication rate of 31.6%. Ghanem et al. [12] investigated the use of 2.3 mm reconstruction bone plates in the treatment of unstable infected mandibular fractures in 32 patients, none of whom showed postoperative evidence of infection or hardware failure. However, in that study, the definition of primary infection was based on delay from injury, and clinical signs of infection were not considered. According to our results, infection complications cannot be completely prevented by reconstruction plate or extraoral approach in clinically infected fractures.

In addition to or even instead of the type of plate, attention should be paid to the length, type, and stability of screws, the accuracy of the reduction, tissue handling during surgery, and the effect of teeth and occlusion on fracture site healing. Thus, the results may be explained by several factors that should be considered in clinical work. Careful infection debridement, optimal fracture reduction, and support for treatment compliance are needed to prevent complications.

**Fig. 2:**
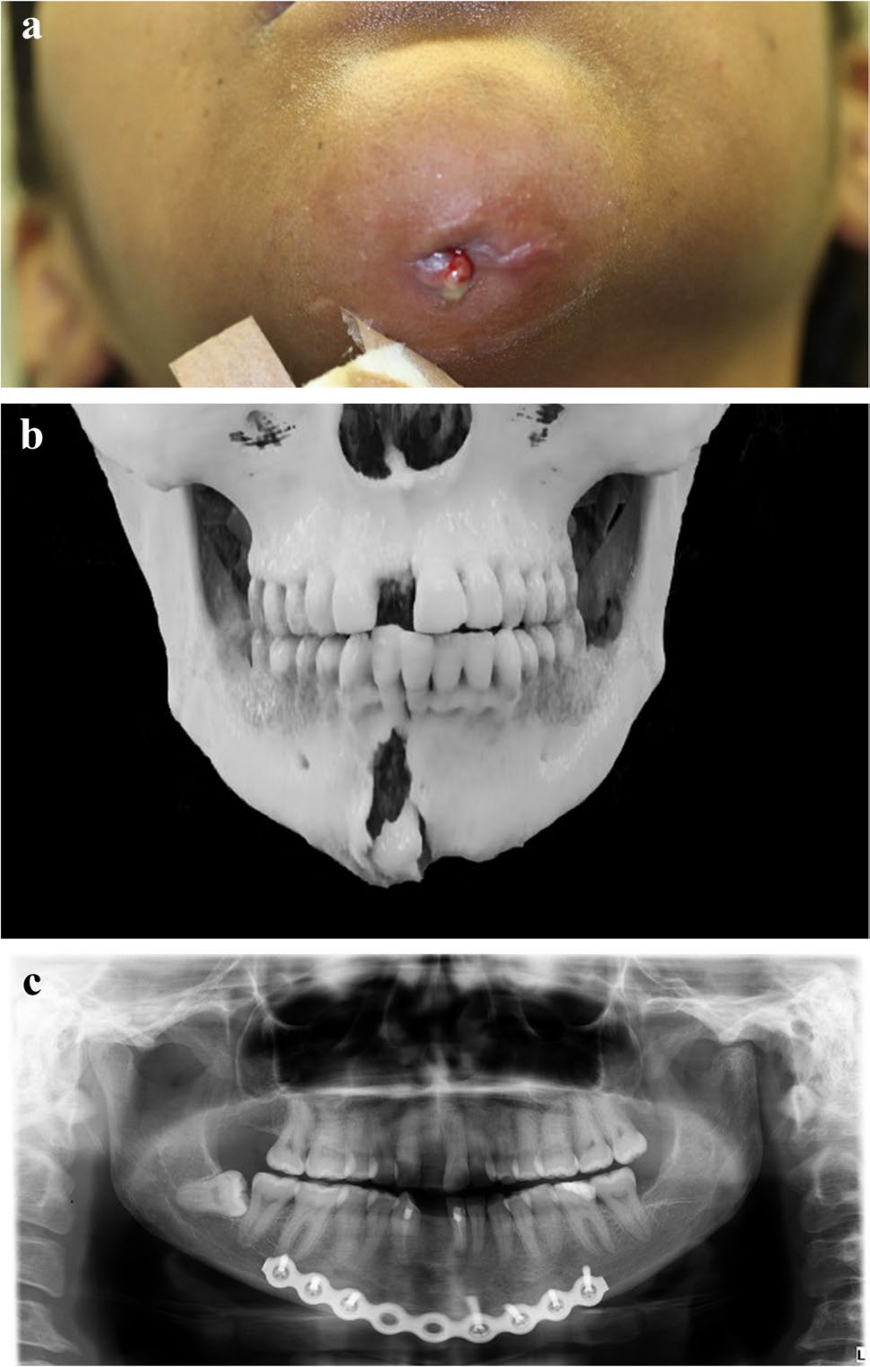
**a** The patient was evaluated for an infected mandibular fracture two months after falling down stairs. A purulent wound formed in the lower jaw after falling, which was treated by medical doctors with two courses of antibiotics. Before ending up in the oral and maxillofacial emergency clinic, the patient also received a blow to her mandible. The patient had an infected symphysis fracture and a fracture in the left condyle. **b** The patient had osteolysis, fragmentation, and chronic infection in the fracture line in the symphysis that can be seen in the preoperative 3D reconstruction of computed tomography. The occlusion was surprisingly in place, but clinically the fracture fragments were mobile, and the symphysis fracture was found to be non-ossified. **c** A reconstruction plate was pre-bent on a 3D-printed model to ensure the comprehensive fit of the reconstruction plate on the lower border of the mandible. Because there were no changes in occlusion, preoperative virtual reduction of the fracture was not required. Extraoral approach was used for osteosynthesis. The reconstruction plate was first fixed in place and removed during careful local debridement of the fracture line. A free iliac crest bone graft was harvested and placed in the area of ​​the mandibular bone defect. Right lower incisors were extracted during the surgery. Teeth that were close to the fracture line required endodontic treatment. Patient developed recurrent infection five weeks postoperatively, which, however, did not affect the ossification process. Patient’s (Fig. 2a) dental panoramic radiograph confirms a favorable recovery process 4.5 months postoperatively. Left condyle fracture healed without surgery

Only one patient received bone grafting in primary fracture surgery (Fig. 2). Benson et al. [13] studied bone grafts in the presence of pus and included 50 infected fractures. They reported that four patients developed recurrent infection, and three had non-union with loss of graft. The only correlating factor with failure was immunocompromising disease. Thus, immediate grafting in infected mandibular fractures seems effective. However, based on our data, surprisingly, mandibular fracture defects associated with infection healed without bone grafting, and non-union was a rare complication.

In infected mandibular fractures, antibiotic therapy is indicated and can be targeted more precisely after specimen culturing. In a previous study [7], alpha and beta-haemolytic streptococci were the most common pathogens found in cultures. Furthermore, viridans streptococci, especially *Streptococcus anginosus*, were the predominant bacteria cultured in our patient population. Empirical antibiotic treatment was started when the patient entered the hospital. Antibiotic treatment was in most cases targeted after the result of bacterial culture. The duration of postoperative antibiotic course varied between 2 and 33 days (mean 11.2 days, median 11 days) and is clearly longer than in mandibular fractures without clinical infection signs prior to surgery [3]. However, complications were not prevented by antibiotic medication or anticipated by anamnesis. Only one patient had type 1 diabetes and had no postoperative complications. Smoking or alcohol/drug abuse did not explain complication rates (Table 3). Future studies should focus on effective local care to prevent primary infection and avoid prolonged antibiotic therapies and recurrent infections.

Limitations of this study comprise its retrospective nature, lack of established protocol in the surgical approach and fixation method selection, and the rather small study population due to the low incidence of these types of fractures. Multicentre studies would provide more scientific evidence of optimal treatment for these patients. In addition, some patients were lost during follow-up, and thus, minor surgical site complications may have gone unrecorded in our data. However, as the department is the region’s only emergency department treating mandibular fractures, patients would have most likely made contact had notable complications occurred.

In conclusion, adequate and early diagnosis of mandibular fractures is essential to reduce the occurrence of infection complications. Both intraoral and extraoral approaches can be successfully used in infected mandibular fractures, but patient selection should be done with care and wound dehiscence risk related to intraoral approach noted. Infected mandibular fractures heal mainly without bone grafting, and non-union is a rare complication. Less severe surgical site complications are common and occur in almost one-third of patients, with a delay of up to three or four months, emphasizing the importance of postoperative follow-up. Because of low treatment compliance and high surgical site complication risk, comprehensive treatment in the primary phase is critical in this patient population.

## Data Availability

The datasets used and/or analysed during the study are available from the corresponding author on reasonable request and in consideration of patients’ privacy.
